# Supporting SARS-CoV-2 Papain-Like Protease Drug Discovery: *In silico* Methods and Benchmarking

**DOI:** 10.3389/fchem.2020.592289

**Published:** 2020-11-05

**Authors:** Tamer M. Ibrahim, Muhammad I. Ismail, Matthias R. Bauer, Adnan A. Bekhit, Frank M. Boeckler

**Affiliations:** ^1^Department of Pharmaceutical Chemistry, Faculty of Pharmacy, Kafrelsheikh University, Kafrelsheikh, Egypt; ^2^Department of Pharmaceutical Chemistry, Faculty of Pharmacy, The British University in Egypt, Cairo, Egypt; ^3^Structure, Biophysics and Fragment-Based Lead Generation, Discovery Sciences, R&D, AstraZeneca, Cambridge, United Kingdom; ^4^Department of Pharmacy, Eberhard-Karls University, Tuebingen, Germany; ^5^Department of Pharmaceutical Chemistry, Faculty of Pharmacy, Alexandria University, Alexandria, Egypt; ^6^Pharmacy Program, Allied Health Department, College of Health and Sport Sciences, University of Bahrain, Zallaq, Bahrain

**Keywords:** COVID-19, docking, VS, benchmarking, DEKOIS 2.0, PLpro

## Abstract

The coronavirus disease 19 (COVID-19) is a rapidly growing pandemic caused by the severe acute respiratory syndrome coronavirus 2 (SARS-CoV-2). Its papain-like protease (SARS-CoV-2 PLpro) is a crucial target to halt virus replication. SARS-CoV PLpro and SARS-CoV-2 PLpro share an 82.9% sequence identity and a 100% sequence identity for the binding site reported to accommodate small molecules in SARS-CoV. The flexible key binding site residues Tyr269 and Gln270 for small-molecule recognition in SARS-CoV PLpro exist also in SARS-CoV-2 PLpro. This inspired us to use the reported small-molecule binders to SARS-CoV PLpro to generate a high-quality DEKOIS 2.0 benchmark set. Accordingly, we used them in a cross-benchmarking study against SARS-CoV-2 PLpro. As there is no SARS-CoV-2 PLpro structure complexed with a small-molecule ligand publicly available at the time of manuscript submission, we built a homology model based on the ligand-bound SARS-CoV structure for benchmarking and docking purposes. Three publicly available docking tools FRED, AutoDock Vina, and PLANTS were benchmarked. All showed better-than-random performances, with FRED performing best against the built model. Detailed performance analysis via pROC-Chemotype plots showed a strong enrichment of the most potent bioactives in the early docking ranks. Cross-benchmarking against the X-ray structure complexed with a peptide-like inhibitor confirmed that FRED is the best-performing tool. Furthermore, we performed cross-benchmarking against the newly introduced X-ray structure complexed with a small-molecule ligand. Interestingly, its benchmarking profile and chemotype enrichment were comparable to the built model. Accordingly, we used FRED in a prospective virtual screen of the DrugBank^1^ database. In conclusion, this study provides an example of how to harness a custom-made DEKOIS 2.0 benchmark set as an approach to enhance the virtual screening success rate against a vital target of the rapidly emerging pandemic.

## Introduction

The latest situation report of the World Health Organization (WHO), of May 6, 2020, reported that COVID-19 is highly spreading worldwide in over 184 countries and responsible so far for >3.6 million cases and >260,000 fatalities. Severe acute respiratory syndrome coronavirus 2 (SARS-CoV-2) is the causative virus for COVID-19 and was recognized in Wuhan, China (Li et al., 2020b; Qian et al., 2020; Rabi et al., 2020; Tilocca et al., 2020). Coronaviruses belong to a large family of enveloped single-stranded RNA genome (ssRNA) that belong to the Coronaviridae family and divided into four genera: alpha, beta, gamma, and delta coronaviruses (Yang and Leibowitz, 2015). Among coronaviruses, some instigated several respiratory diseases, such as SARS-CoV (Drosten et al., 2003), Middle East respiratory syndrome coronavirus (MERS-CoV) (Zaki et al., 2012), and the pandemic COVID-19 (Rabi et al., 2020). SARS-CoV-2 are beta coronaviruses (Li et al., 2020a; Rabi et al., 2020) with symptoms usually resembling other respiratory virus infections like influenza and rhinovirus (Hsih et al., 2020).

Upon the virion entry to the host cell, translation of 5′-terminal open reading frames (ORF1a and ORF1ab) is initiated to produce two large polyproteins, pp1a and pp1ab, which are then processed by papain-like protease (PLpro) and 3C-like protease (3CLpro), also called main protease (Mpro) (Barretto et al., 2005; Mielech et al., 2015). This processing is crucial for the release of 16 non-structural proteins (nsps1–16). The formation of the replicase complex essential for viral genome replication is dependent on nsps (Fehr and Perlman, 2015). PLpro plays an essential role for the release of nsp1–3 from the viral polyprotein which are indispensable for viral replication. Also, PLpro has been observed to negatively regulate the host innate immune response toward the viral infection by its deubiquitinating and deISGylating effect (Báez-Santos et al., 2015; Clemente et al., 2020). As a result, PLpro has been recognized as an important target for viral replication suppression endeavors in SARS-CoV and SARS-CoV-2 (Báez-Santos et al., 2015; Freitas et al., 2020).

Structure-based virtual screening (SBVS) remained a crucial technique in modern drug discovery (Schapira et al., 2003; Schneider, 2010; Santiago et al., 2012; Scior et al., 2012). Molecular docking is widely employed in SBVS campaigns, which exploits the structural information of the molecular targets binding sites to assess large molecular databases and predict the preferred binding of compounds prior to the biological screening. Nevertheless, the docking tool and the VS workflow selection must be assessed using benchmarking molecular sets. The benchmarking depends on challenging the VS workflow to enrich known bioactives within a set of decoys (Bauer et al., 2013; Ibrahim et al., 2015a).

In this study, we benchmark three publicly available docking tools, AutoDock Vina, PLANTS, and FRED against SARS-CoV-2 PLpro. One challenge comprises the absence of small molecules known to inhibit SARS-CoV-2 PLpro and consequently to generate a matching decoy set. Another challenge encompasses the absence of structural conformation of the SARS-CoV-2 PLpro binding site when complexed with conventional small molecules. To overcome these challenges, we conducted a cross benchmark by generating a DEKOIS 2.0 benchmark set of known SARS-CoV PLpro bioactives for SARS-CoV-2 PLpro, using the advantage of the high similarity between both enzymes and identical binding site residues. Furthermore, we modeled the conformation of SARS-CoV-2 complexed with small molecule based on its co-crystal structure homolog, SARS-CoV PLpro. Guided by the benchmarking outcome, we performed a VS effort against the DrugBank database and discuss the most promising hits. This study offers an example of how to employ a DEKOIS 2.0 benchmark set to enhance virtual screening success against a vital target of SARS-CoV-2. This procedure may facilitate virtual finding also against other rapidly resolved protein structures of SARS-CoV-2.

## Results and Discussions

### Multiple-Sequence Alignment and Modeling

**Figure 1:**
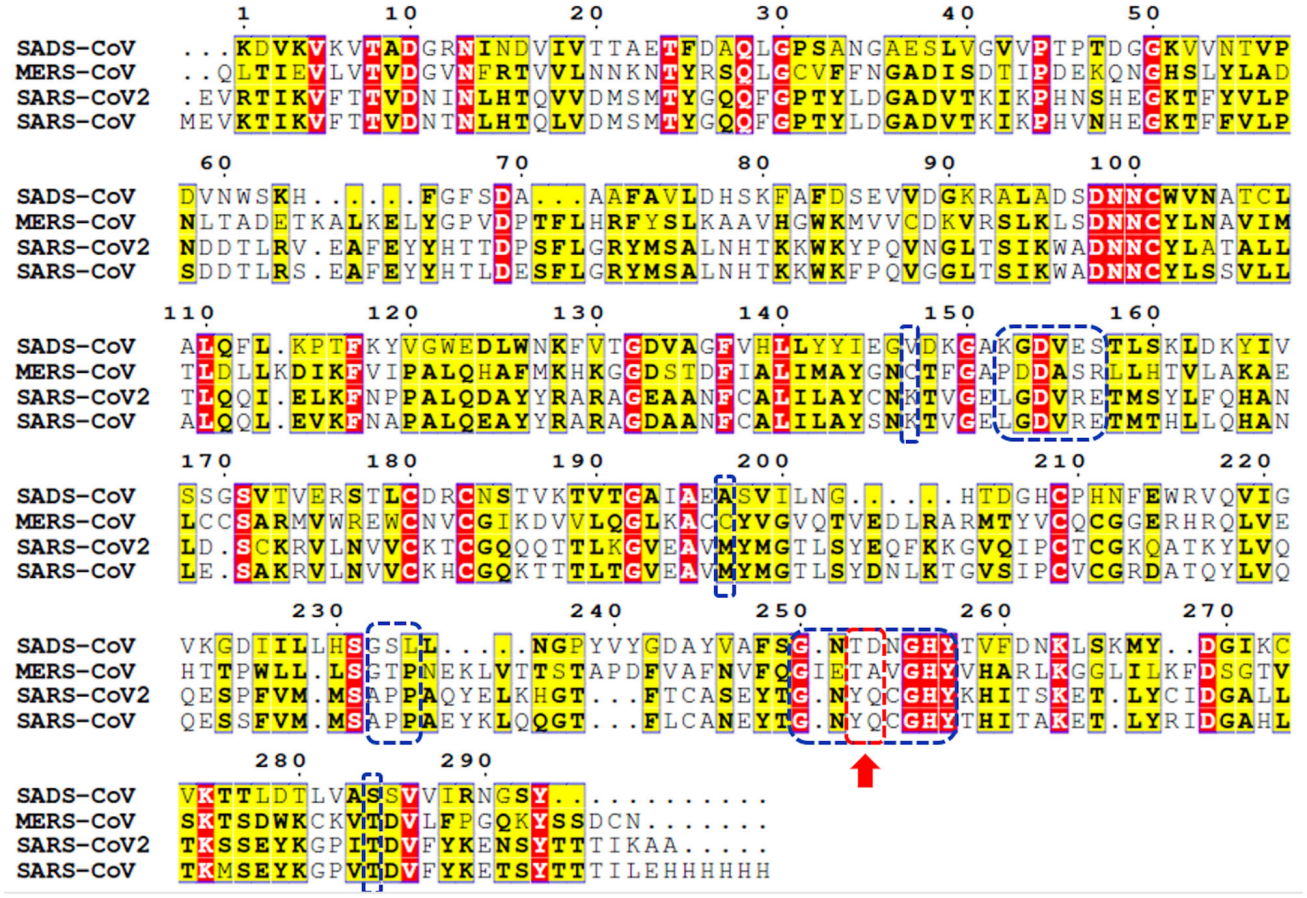
Multiple-sequence alignment for PLpro sequences of some clinically relevant corona virus strains (SADS, MERS, SARS-CoV-2, and SARS-CoV). Identical and less conserved residues are highlighted red and yellow, respectively. Red arrow and red-dashed rectangle indicate the flexible loop residues Tyr269 and Gln270 as a part of the binding site. Residues of the binding site are marked by blue-dashed rectangles.

Genome sequencing showed an 80% similarity between SARS-CoV and SARS-CoV-2 genetic sequences (Rabaan et al., 2020; Rabi et al., 2020; Zhou et al., 2020). The multiple-sequence alignment (MSA) of PLpro from the most clinically relevant human corona viruses, e.g., SADS, MERS, SARS-CoV-2, and SARS-CoV PLpro, is portrayed in Figure 1. Comparing the percentage sequence identity of SARS-CoV-2 PLpro with other human corona viruses reveals that SARS-CoV PLpro is the closest strain to the SARS-CoV-2 PLpro with 82.9% identity. Interestingly, SARS-CoV PLpro and SARS-CoV-2 PLpro share identical binding site residues for small-molecule binding, as marked by the blue-dashed rectangle in Figure 1. Residues Tyr269 and Gln270 in SARS-CoV, marked by the red-dashed rectangle, play an important role in small molecule-protein binding event (Ratia et al., 2008; Ghosh et al., 2009). They encompass a flexible loop capable of accommodating different backbone and side chain conformations. Interestingly, it was reported that small-molecule inhibitors of SARS-CoV PLpro were not able to recognize and specifically inhibit MERS-PLpro (Lee et al., 2015). This is attributed to many factors among which is the lack of the key residues Tyr269 and Gln270 of SARS-CoV PLpro in MERS-CoV PLpro (Lee et al., 2015), as shown by the red arrow of Figure 1. Interestingly, such key residues are present in SARS-CoV-2 PLpro (Tyr268 and Gln269).

### Structural Aspects

#### SARS-CoV PLpro vs. SARS-CoV-2 PLpro

**Figure 2:**
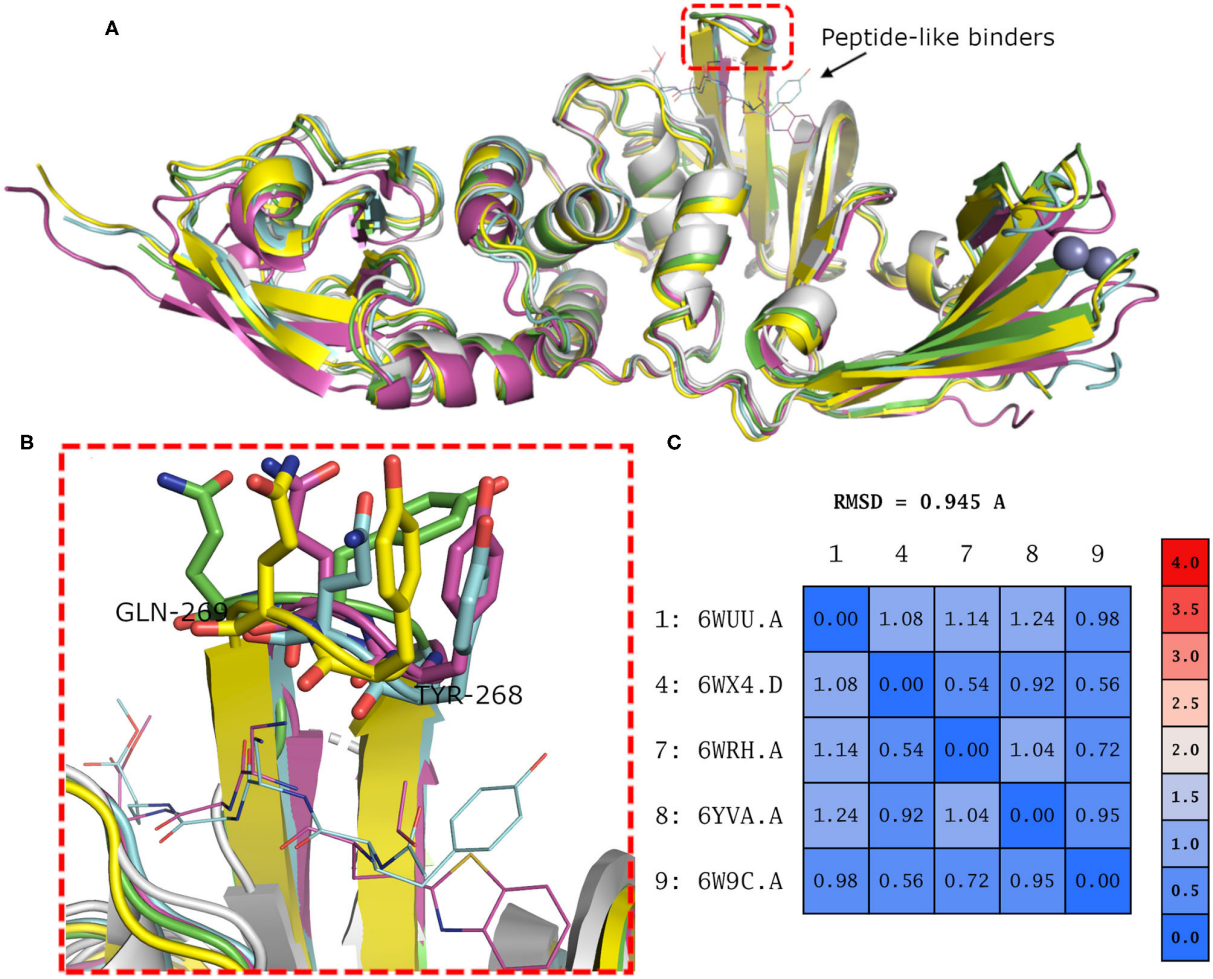
**(A)** Superposition of the different SARS-CoV-2 PLpro structures including three apo structures and two complexed with peptide binders, namely, PDB ID: 6W9C, 6WRH, 6YVA, 6WUU, and 6WX4 as green, yellow, simon, purple, and gray cartoon representation, respectively. **(B)** Different conformations of the backbone and side chains of the key Tyr268 and Gln269 residues. **(C)** Pairwise RMSD matrix for all structures calculated for their α carbon atoms.

Binding site residues of both SARS-CoV and SARS-CoV-2 PLpro are 100% identical. The PDB structures of both (SARS-CoV and SARS-CoV-2 PLpro) proteins appear to show a comparable fold and do not deviate substantially in backbone conformations. For SARS-CoV-2 PLpro, three crystal structures are available in the apo form, with an average pairwise RMSD matrix for the backbone around 1 Å, as seen in Figure 2. The binding site exhibits more diverse conformations for the backbone and side chains of the flexible loop (i.e., Tyr268 and Gln269) where the side chains mostly appear to point outward to the solvent exposed area, as shown in Figure 2B. These conformations represent only the unbound state (apo state) for the binding site. It is noteworthy that some SARS-CoV-2 PLpro structures complexed with peptide-like binders were introduced in the PDB, while none complexed with conventional small molecules are available yet at the time of manuscript submission. Figure 2 shows a depiction of these SARS-CoV-2 PLpro structures.

**Figure 3:**
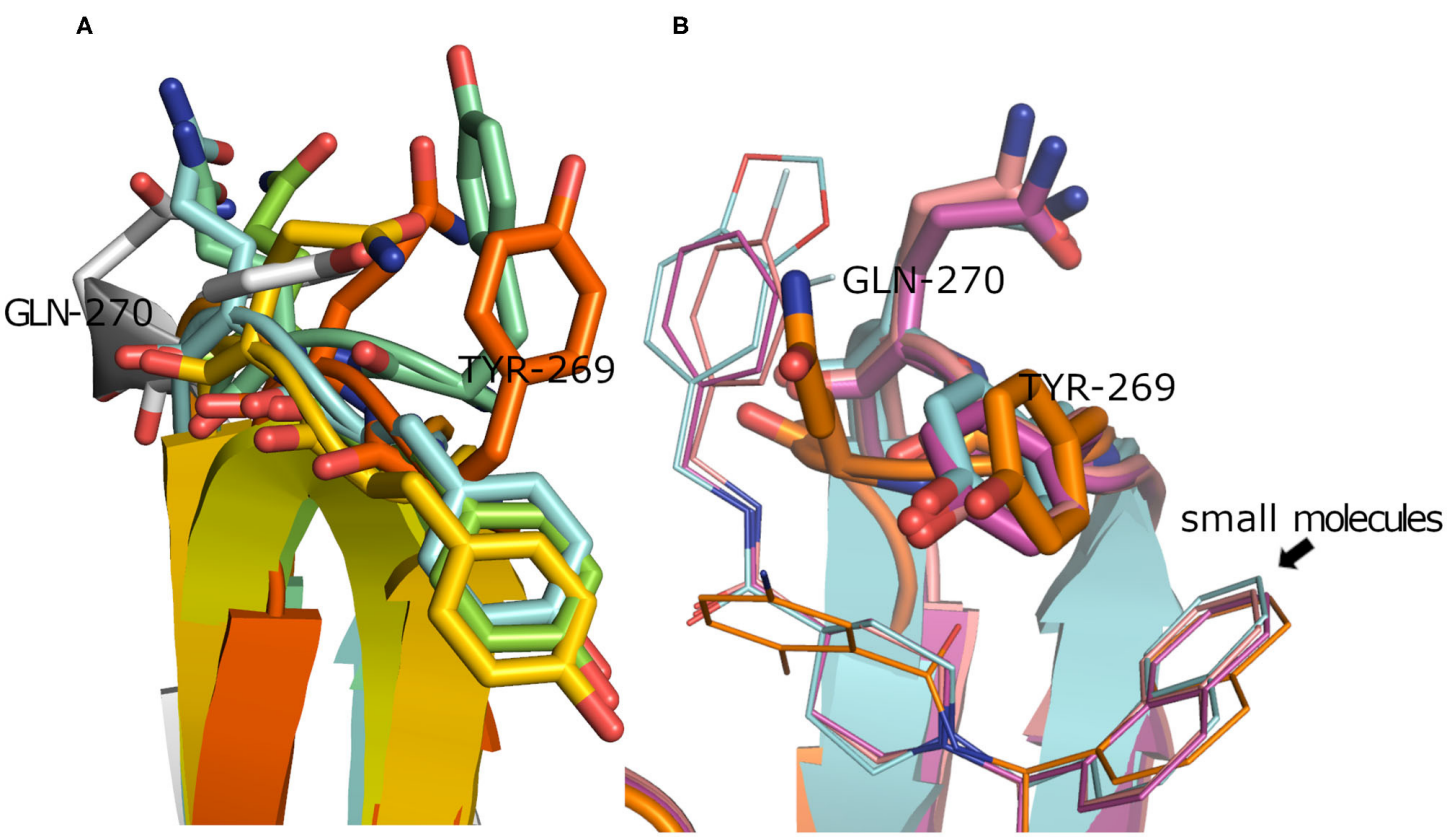
**(A)** Superposition of the SARS-CoV PLpro *apo* structures for PDB ID: 4M0W (Chou et al., 2014), 4MM3 (Ratia et al., 2014), 5E6J (Bekes et al., 2016), 5TL6 (Daczkowski et al., 2017), 5TL7 (Daczkowski et al., 2017), 5Y3Q (Lin et al., 2018) (conjugated with beta-mercaptoethanol), and 2FE8 (Ratia et al., 2006) as cyan, pale blue, green, yellow, orange, dark orange, and gray cartoon representation, respectively, showing vast conformations of Tyr269 and Gln270 residues. **(B)** Superposition of the SARS-CoV PLpro co-crystal structures with small molecules for PDB ID: 3MJ5 (Ghosh et al., 2010), 4OVZ (Baez-Santos et al., 2014), 4OW0 (Baez-Santos et al., 2014), and 3E9S as cyan, pale violet, purple, and gold cartoon representation, respectively, exhibiting more ordered conformations of Tyr269 and Gln270 residues.

To have a clue on the possible rearrangement of SARS-CoV-2 PLpro upon conventional small-molecule binding, we investigated its homolog, the SARS-CoV PLpro co-crystal structures complexed with small molecules. For this, we retrieved 11 high-quality crystal structures of SARS-CoV PLpro for both small-molecule complexes and the unbound structures to small molecules (referred to as apo structures in this study). Like SARS-CoV-2, the apo SARS-CoV PLpro (seven crystal structures) displayed a wide range of conformations for the backbone and side chains of the flexible loop (residues Tyr269 and Gln270 in Figure 3A) with an average pairwise RMSD values for the backbone <2 Å (data not shown). On the other hand, the co-crystal structures with small molecules (four crystal structures) showed more ordered rearrangement of Tyr269 for all of them. This is likely to offer a hydrophobic wall for optimum interactions with the aromatic substructure of the bound small molecule (Figure 3B; Lee et al., 2015), while Gln270 appeared to adapt more conformations depending on ligand topology and size. Based on the previous, it is likely that SARS-CoV-2 PLpro would behave in a similar fashion to its analog, SARS-CoV PLpro, upon small-molecule binding. Therefore, for docking and benchmarking purposes, and due to the lack of co-crystallized structures of SARS-CoV-2 PLpro with small molecules, we constructed a homology model for SARS-CoV-2 PLpro complexed with small molecule, based on its co-crystallized homolog SARS-CoV PLpro.

#### Homology Model

**Figure 4:**
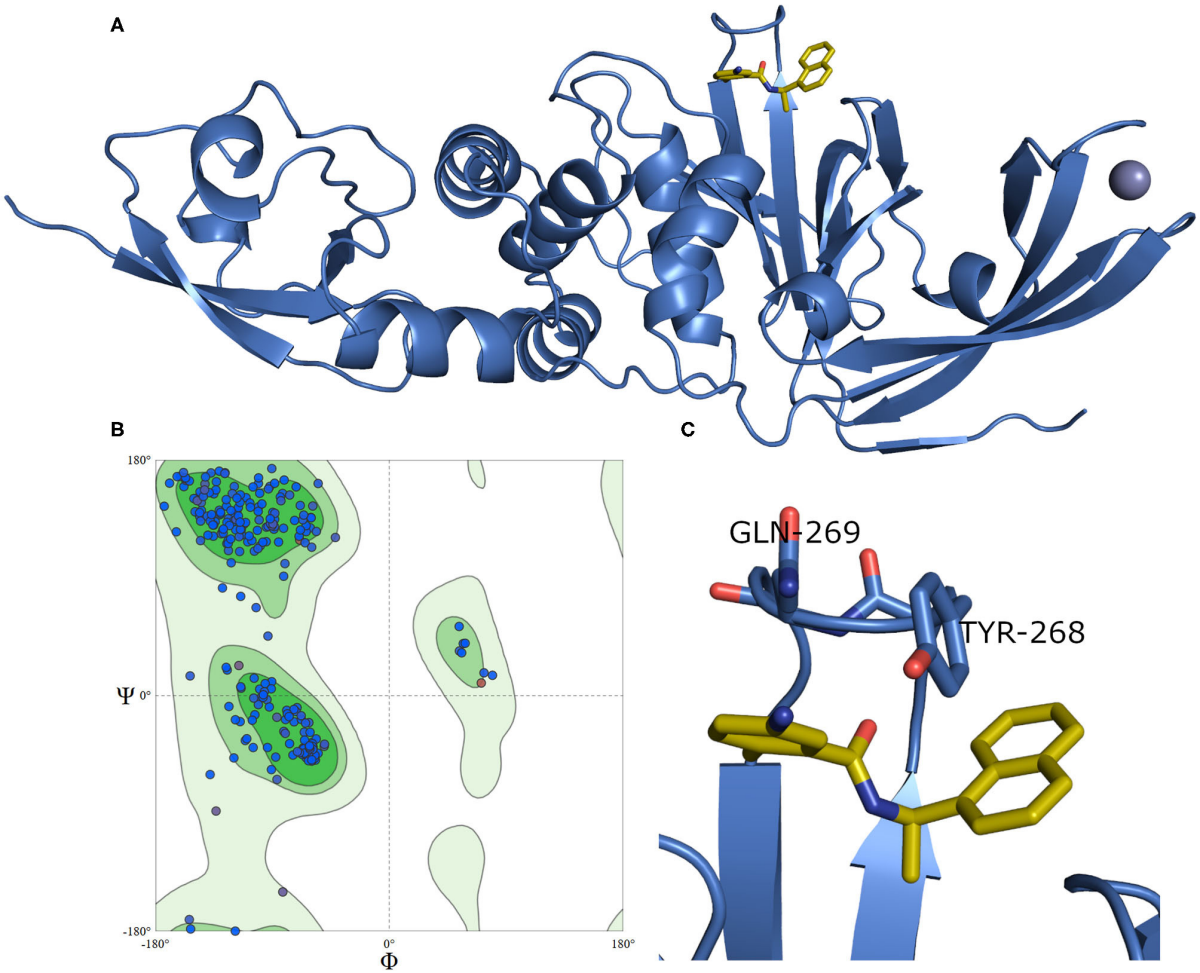
**(A)** The newly emerged SARS-CoV-2 PLpro ligand-protein-complexed model built by SWISS-MODEL in the blue cartoon and its bound ligand in gold sticks. **(B)** Ramachandran plot for SARS-CoV-2 PLpro model. **(C)** The enlarged part of the binding site showing the conformations of the key Tyr268 and Gln269 residues.

A model of SARS-CoV-2 PLpro (317 residues) complexed with a small-molecule ligand (compound **TTT**, “5-amino-2-methyl-N-[(1R)-1-naphthalen-1-ylethyl]benzamide”) is built by the aid of the automated homology modeling, SWISS-MODEL (Waterhouse et al., 2018) web server, using SARS-CoV PLpro (PDB ID: 3E9S, chain A) as a template, as shown in Figure 4. The model has a high sequence identity (82.9%) to the template. Quality estimates for the built model indicated high reliability of the model, with a QMEAN (Benkert et al., 2011) value of −0.22 and GMQE (Global Model Quality Estimation) (Waterhouse et al., 2018) value of 0.95. The Ramachandran plot, in Figure 4B, shows that 100% of the residues are in the allowed regions. Also, it displayed that 94.9% of the residues, including the binding site residues, are in the most favored region. In addition, the validation web servers (SAVES, 2020) presented that 98.73% of the residues have averaged 3D-1D score ≥0.2 on the Verify 3D module. The overall quality factor of ERRAT is 92.8 %. Globally, these values indicate a valid and a high-quality homology model.

Figure 4C exhibits a noticeable difference in the side chain conformations of the key Tyr298 and Gln269 between the model and the X-ray structure complexed with a peptide-like inhibitor (Figure 2B). Unlike the model, both key residues of the latter structure (i.e., PDB ID: 6WUU) appear to point outward to the solvent-exposed area.

**Figure 5:**
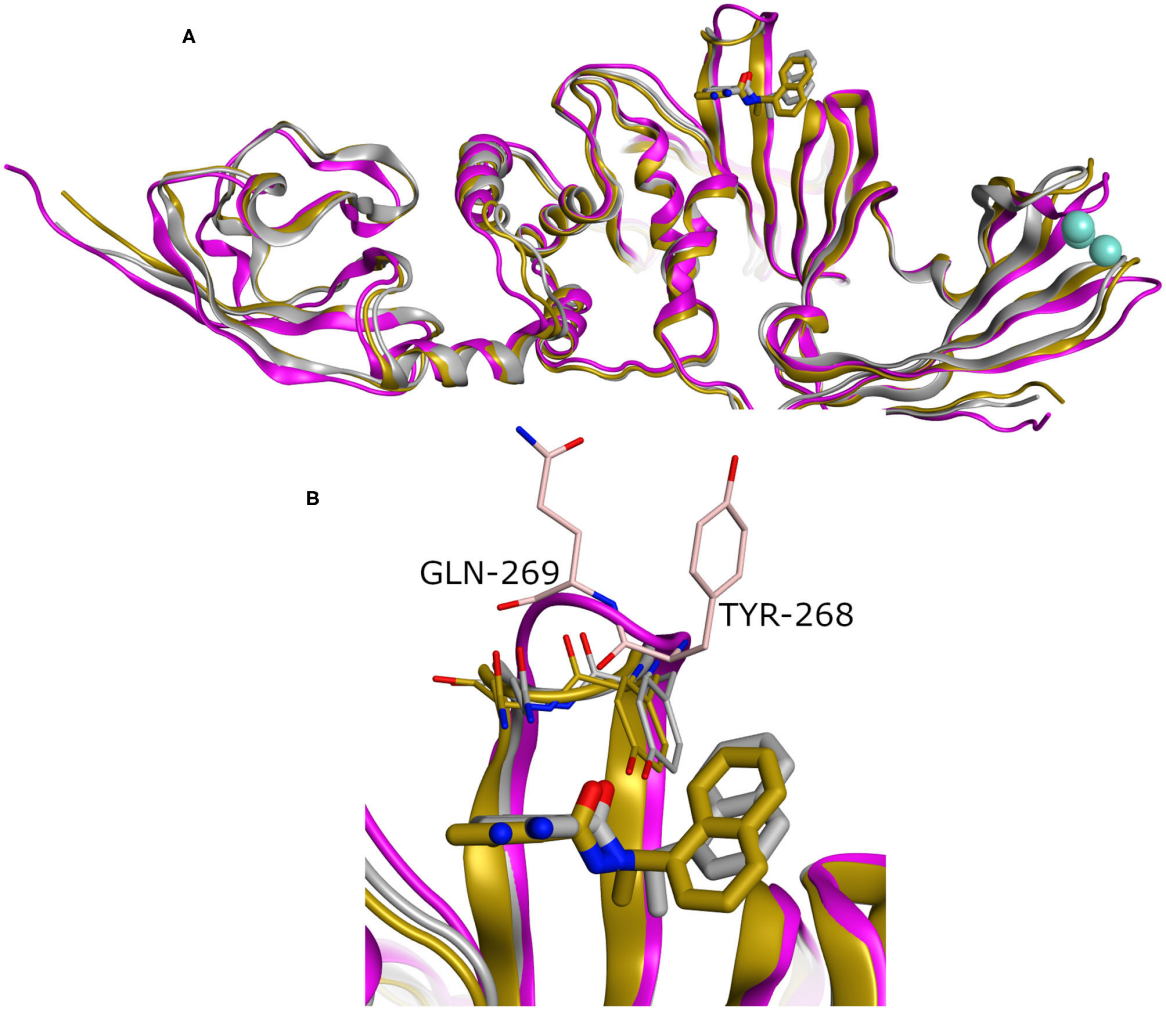
**(A)** Superposition of three SARS-CoV2 PLpro structures involving the homology model, the X-ray structure complexed with peptide-like inhibitor (PDB ID: 6WUU), and the recently introduced X-ray structure complexed with **TTT** (PDB ID: 7JRN) as gray, purple, and gold cartoon representation, respectively. **(B)** The enlarged part of the binding site showing comparable conformations of the key Tyr268 and Gln269 residues and the bound ligand (**TTT)** for both the homology and the X-ray structure (PDB ID: 7JRN). The co-crystal peptide-like ligand (PDB ID: 6WUU) was omitted for clarity.

While our manuscript was under review, new X-ray co-crystal structures with impact on our study were released. Thus, we closely investigated an example of these structures in comparison to the homology model we built. For instance, we considered the recently introduced X-ray co-crystal structure complexed with compound **TTT** (PDB ID: 7JRN). This structure is for the wild type and with best resolution available for a SARS-CoV2 PLpro-**TTT** complex. We did not observe a significant difference between both the model and the X-ray structure (average RMSD for the whole proteins = 0.98 Å). Interestingly, unlike the X-ray structure complexed with the peptide-like inhibitor (PDB ID: 6WUU), both the homology model and the SARS-CoV2 PLpro-**TTT** complex (PDB ID: 7JRN) exhibited almost similar conformations for the key residues Tyr298 and Gln269, as well as for the pose of **TTT**, as shown in Figure 5. This reflects the high reliability and quality of our predicted model.

### Benchmarking

Generally, it was reported that VS performance depends strongly on the respective target properties (Bauer et al., 2013). Accordingly, diverse docking methods and scoring schemes may work better on some targets than others. To avert delays and unnecessary efforts on unproductive VS strategies, it is crucial to evaluate the performance of different VS setups in order to select the most effective workflow (Ibrahim et al., 2015a). Screening performance can be assessed using molecular benchmark sets, such as DEKOIS 2.0 (Vogel et al., 2011; Bauer et al., 2013; Boeckler et al., 2014) and DUD-E (Mysinger et al., 2012). The idea aims at recognizing the suitable docking tool that can efficiently differentiate between the bioactive ligands and the generated challenging decoys. The higher the number of bioactives at the top of the score-ordered list of screened molecules, the better is the respective screening performance.

Due to the lack of small-molecule ligands for SARS-CoV-2 PLpro, and the high similarity of both SARS-CoV and SARS-CoV-2 PLpro enzymes, we performed cross benchmarking of SARS-CoV-2 PLpro based on SARS-CoV PLpro reported small-molecule ligands. We generated a challenging decoy set by our DEKOIS 2.0 (Vogel et al., 2011; Bauer et al., 2013; Boeckler et al., 2014) protocol from the available bioactives of SARS-CoV PLpro (retrieved from BindingDB; Liu et al., 2007). Then, we conducted a benchmarking study using three publicly available docking tools, namely, AutoDock Vina, PLANTS, and FRED. Pleasingly, a recent study by Freitas et al. confirmed our cross benchmarking approach since the naphthalene-based SARS-CoV PLpro inhibitors showed inhibitory activities against SARS-CoV-2 PLpro and stopped the SARS-CoV-2 replication (Freitas et al., 2020).

**Figure 6:**
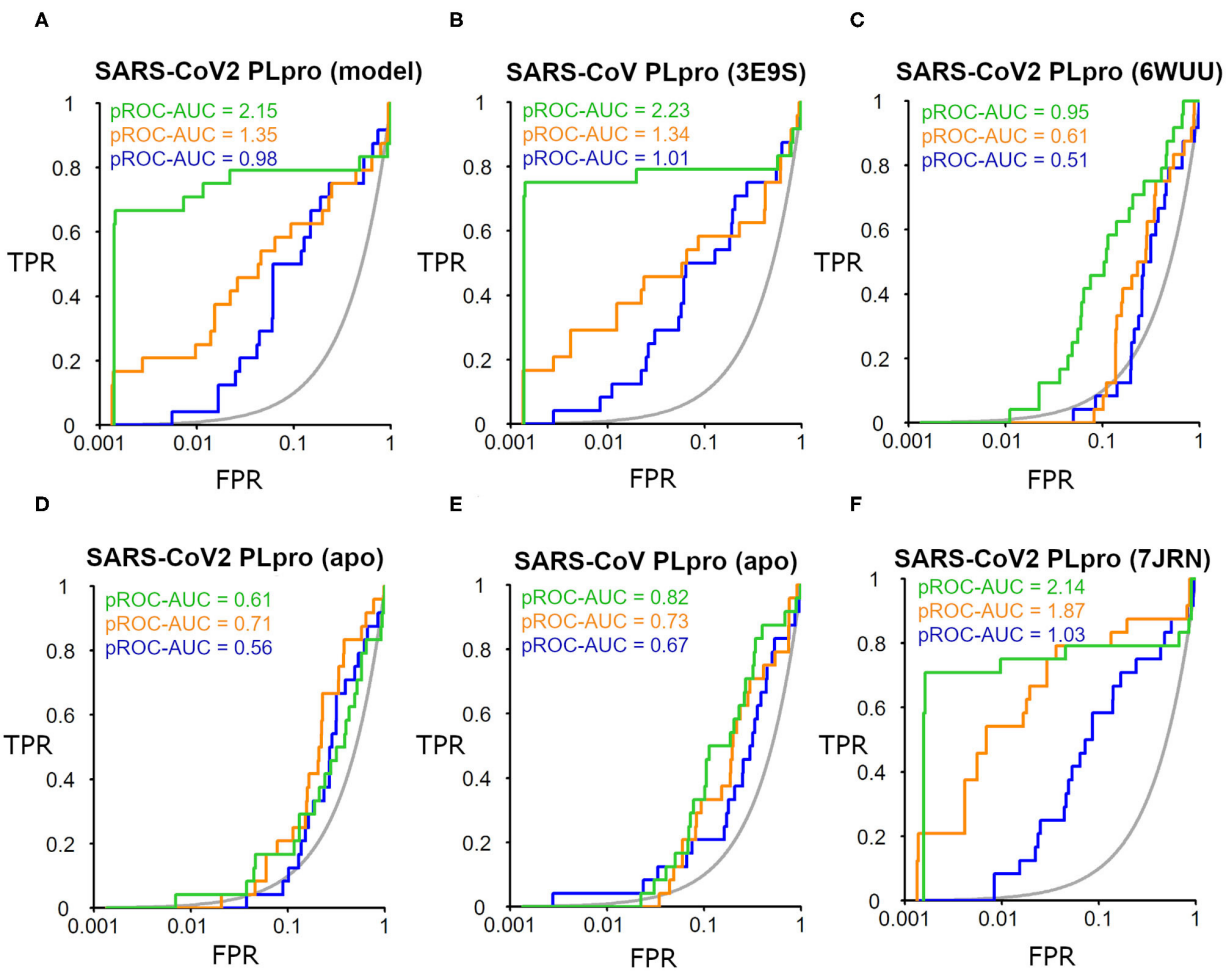
pROC plots of docking experiments showing the screening performance of both ligand-bound conformations of the SARS-CoV-2 PLpro model, the co-crystal structure of SARS-CoV PLpro (PDB ID: 3E9S), and the co-crystal structure SARS-CoV2 PLpro (PDB ID: 6WUU) as **(A–C)**, respectively. **(D,E)** are for the screening performance of both *apo* structures of SARS-CoV-2 PLpro (PDB ID: 6W9C) and of SARS-CoV PLpro (PDB ID: 2FE8), respectively. **(F)** The screening performance of the recently introduced X-ray SARS-CoV-2 PLpro complexed with **TTT** (PDB ID: 7JRN). The graphs of the docking tools FRED, AutoDock Vina, and PLANTS screening results are shown in green, orange, and blue, respectively. The true positive rate (TPR) is the fraction of recovered bioactives; the false-positive rate (FPR) is the fraction of recovered decoys from a score-ordered list of all decoys. The gray line corresponds to a random screening performance.

The SARS-CoV-2 PLpro homology model benchmarking results revealed that FRED screening performance exhibited the best performance with a pROC-AUC value of 2.15, compared to pROC-AUC values of 1.35 and 0.98 for AutoDock Vina and PLANTS, respectively (Figure 6A). Interestingly, the screening performance against the co-crystal SARS-CoV PLpro structure (PDB ID: 3E9S) yielded a comparable outcome for FRED, and non-significant differences (i.e., ΔpROC-AUC values ≤ 0.05; Bauer et al., 2013) for AutoDock Vina and PLANTS docking tools (Figure 6B). Therefore, these results emphasize also the druggability of the SARS-CoV-2 PLpro homology model by the SARS-CoV PLpro benchmark set. Interestingly, all docking tools exhibited better-than-random performance, i.e., pROC-AUC value >0.43.

In addition, benchmarking results of the co-crystal X-ray structure of SARS-CoV2 with a peptide-like inhibitor (e.g., PDB ID: 6WUU) emphasizes that FRED screening performance appeared to be superior to AutoDock Vina and PLANTS, with pROC-AUC values of 0.95, 0.61, and 0.51, respectively (Figure 6C). Nonetheless, in this case, the three docking tools exhibited significant lower performances compared to the homology model. This is likely attributed to the differences in the backbone and side-chain conformations of the key Tyr298 and Gln269 between the model and the X-ray structure complexed with a peptide-like inhibitor (as shown earlier in Figure 5). We also assessed the *in silico* druggability of the unbound conformation (i.e., apo form) of the binding site of SARS-CoV-2 PLpro (PDB ID: 6W9C) and SARS-CoV PLpro (PDB ID: 2FE8) using the generated DEKOIS 2.0 benchmark set. Most of the docking tools showed significant lower performance compared to the bound state as shown in Figures 6D,E.

The benchmarking outcome of the newly introduced X-ray complexed with **TTT** (PDB ID: 7JRN) displayed non-significant differences from the homology model for FRED and PLANTS, and a slightly improved performance for AutoDock Vina, as seen in Figure 6F. This is likely attributed to the comparable conformations of the key residues (Tyr298 and Gln269) for both protein structures.

**Figure 7:**
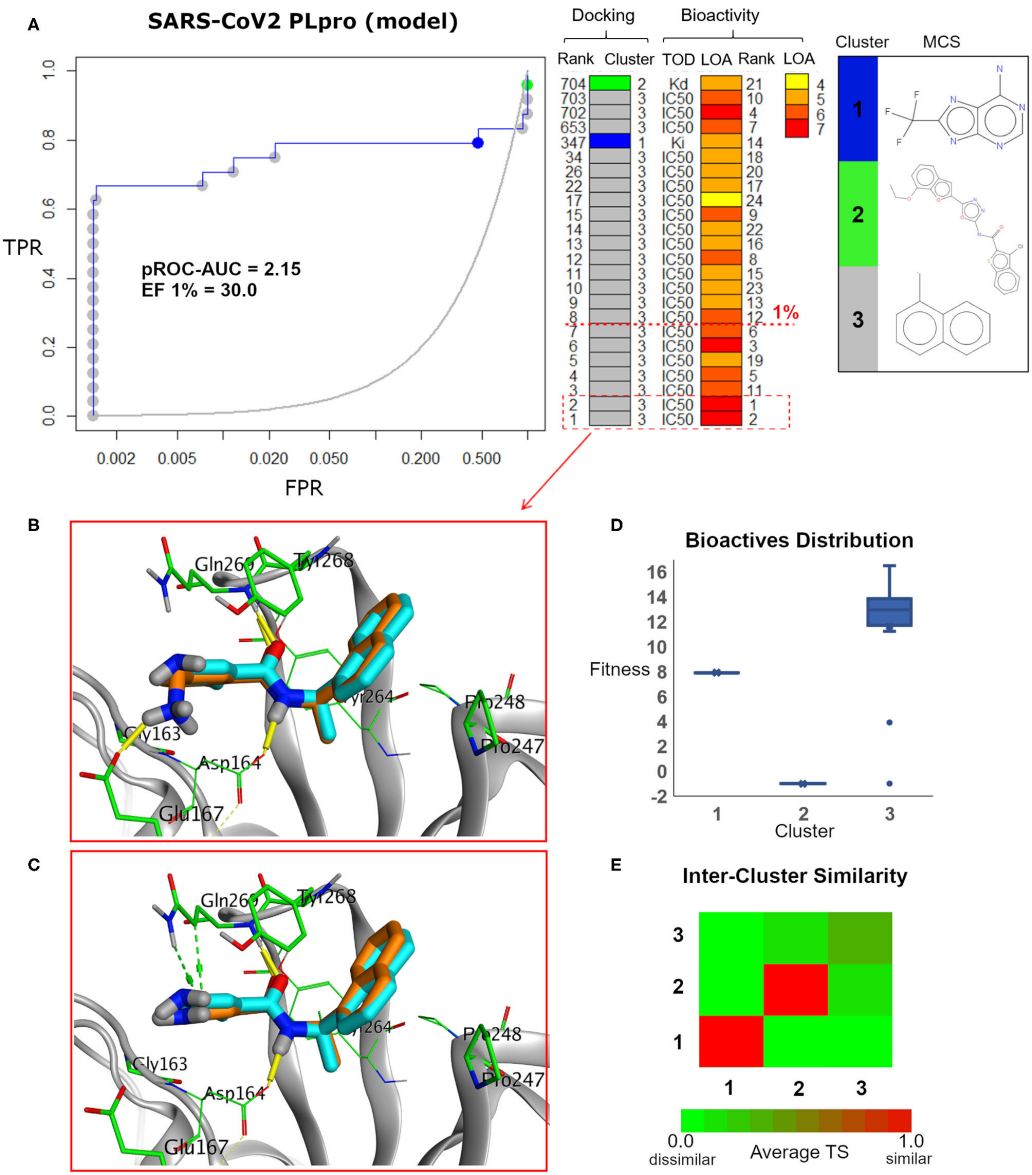
**(A)** pROC-Chemotype plot (Ibrahim et al., 2015b) of the SARS-CoV-2 PLpro model using the FRED docking tool. The docking information is matched with the chemotype represented by the cluster number and the bioactivity information. The bioactivity information is represented as the rank of the ligand bioactivity, where the color scales from yellow (less potent) to red (more potent). The red-dashed line indicates an enrichment of bioactives as at the 1% database. Enrichment Factor (EF) evaluates the capability of the docking tool to find true positives in the score-ranked list compared to the random selection. EF is calculated based on the succeeding equation (Wei et al., 2002) $EF=\frac{Bioactives_{subset}}{N_{subset}}/\frac{Bioactives_{total}}{N_{total}}$. **(B,C)** The best docking and the second-best docking poses of the bioactive set overlaid on the model ligand as orange and cyan sticks, respectively. **(D)** Box plot of the fitness vs. chemotype clusters illustrating the bioactive molecules distribution. Fitness is expressed as the FRED score multiplied by −1 for comparison purposes. **(E)** Heat map of the three chemotype clusters of the SARS-CoV PLpro benchmark set based on the average Tanimoto similarity (Ts) over all cross-cluster pairs. The color gradient represents changes in the average Ts. Green indicates maximum dissimilarity (Ts ≈ 0), and red indicates maximum similarity (Ts = 1).

We analyzed the chemotype enrichment with the “pROC-Chemotype” (Ibrahim et al., 2014, 2015b) plot (see Figure 7) for the benchmarking of the SARS-CoV-2 PLpro model using the FRED docking tool. Only 32 small-molecule binders to SARS-CoV PLpro were introduced and collected by the BindingDB repository (Liu et al., 2007) when searching with the keyword “SARS coronavirus papain-like protease.” These molecules were collected mainly from Ghosh et al. (2009) and Lee et al. (2015). This ended up to 24 small-molecule binders after we curated them and removed the duplicates.

Maximum common substructure (MCS) (Ibrahim et al., 2015b) chemotype clustering demonstrates 3 main clusters representing different chemotype classes. Clusters 1 and 2 represent singletons (i.e., a compound per cluster), while cluster 3 (methyl naphthalene substructure) represents the rest of the bioactive compounds. Therefore, the average *Tanimoto similarity* (Ts) was determined by using definition 1 for clusters 1 and 2, while showing Ts <1 for cluster 3, as shown in the relative intercluster (dis)similarity (Figure 7E). Generally, such MCS clustering behavior reflects the narrow diversity of the known chemotypes, emphasizing the need of developing more diverse small-molecule inhibitors for SARS-CoV PLpro and eventually for SARS-CoV-2 PLpro. The bioactivity data are represented by *level of activity* (LOA) ranging from 10^−4^ to 10^−7^ M and recorded as IC_50_, K_i_, or K_d_ as a *type of data* (TOD), as seen in Figure 7.

The pROC-Chemotype plot visualized that the applied docking protocol is likely capable of detecting high-affinity binders at early enrichment, as seen in Figure 7. For instance, the best two docked active molecules (docking rank 1 and 2) are also the highest in bioactivity (i.e., with bioactivity rank 2 and 1, respectively, Figure 7A) with IC_50_ values of 230 and 460 nM against SARS-CoV PLpro (Ghosh et al., 2009; Lee et al., 2015). Visualizing their docking poses emphasizes that they reproduced the key interactions of the model ligand, as shown in Figures 7B,C. It is worth mentioning that such model ligand (**TTT**) is included in the bioactive set with bioactivity rank 1 and docking rank 2, as shown in Figure 7C. Furthermore, at 1% of the score-ordered database, only bioactive molecules were enriched and none of the decoys were recognized, resulting in an Enrichment Factor (EF 1%) of 30.0. This highlights promising enrichment power for the tool under investigation for such a target.

Figure 7D shows the docking fitness distribution of the bioactive compounds. The docking score ranges from −16.51 (best score) to 1.00 (worse score) and presented as fitness values of 16.51 to −1.00 in Figure 7D. Also, the majority of cluster 3 compounds lie in the superior region of fitness (i.e., fitness >12). Such superior scores can be attributed mainly to the fact that the naphthyl substructures of their docking poses are involved in hydrophobic interactions and packed between the side chain of the key residue Tyr268 and the side chains of Pro247 and Pro248, as seen in e.g., Figures 7B,C.

**Figure 8:**
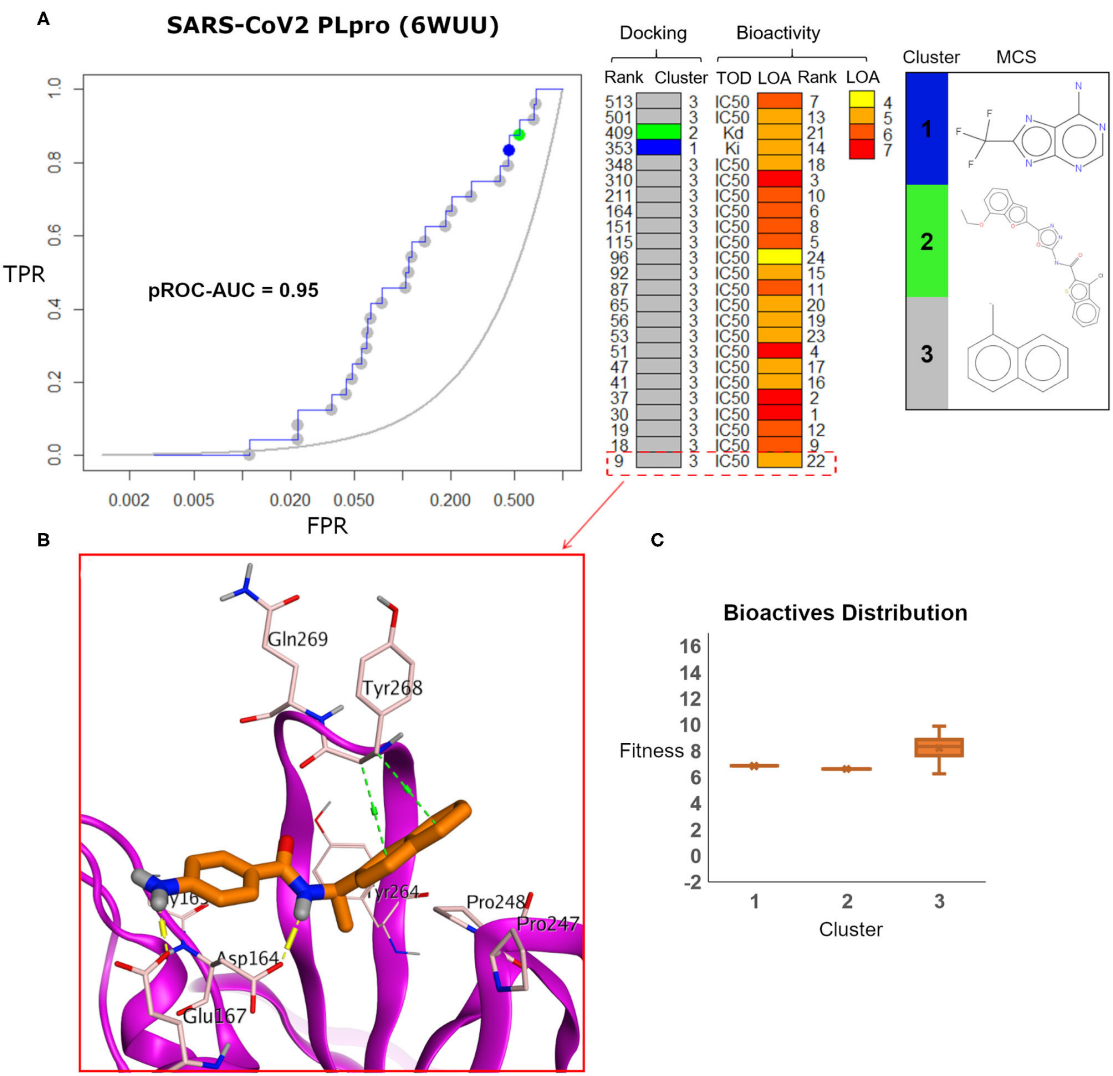
**(A)** pROC-Chemotype plot of SARS-CoV-2 PLpro (PDB ID: 6WUU) using the FRED docking tool. The docking information is matched with the chemotype represented by the cluster number and the bioactivity information. **(B)** The best enriched bioactive compound in the binding site of the protein. **(C)** Box plot of the fitness vs. chemotype clusters illustrating the bioactive molecules distribution.

Visualizing the benchmarking results for the experimental X-ray co-crystal structure (e.g., PDB ID: 6WUU), Figure 8 displays the pROC-Chemotype plot using FRED docking. Unlike the high value of EF 1% for the SARS-CoV2 model (Figure 7), the screening performance of the X-ray co-crystal structure did not enrich any bioactive compounds at 1% of the database. Unlike the best enriched bioactive compounds for the SARS-CoV2 model (Figures 7B,C), the best enriched bioactive (Figure 8B) appeared to lose some contacts with the side chains of the key Tyr268 and Gln269 where their side chains appear to be solvent-exposed and directed outward.

Furthermore, the docking fitness distribution of the bioactive compounds in this case is narrower with inferior score range compared to the model performance. For instance, the docking score ranges from −9.89 (best score) to −6.23 (worst score) and presented as fitness values of 9.89–6.23 in Figure 8C. In this case, molecules of cluster 3 did not gain significant advantage since side chains of the key residues Tyr268 and Gln269 are not likely able to optimally interact with their naphthyl substructures.

**Figure 9:**
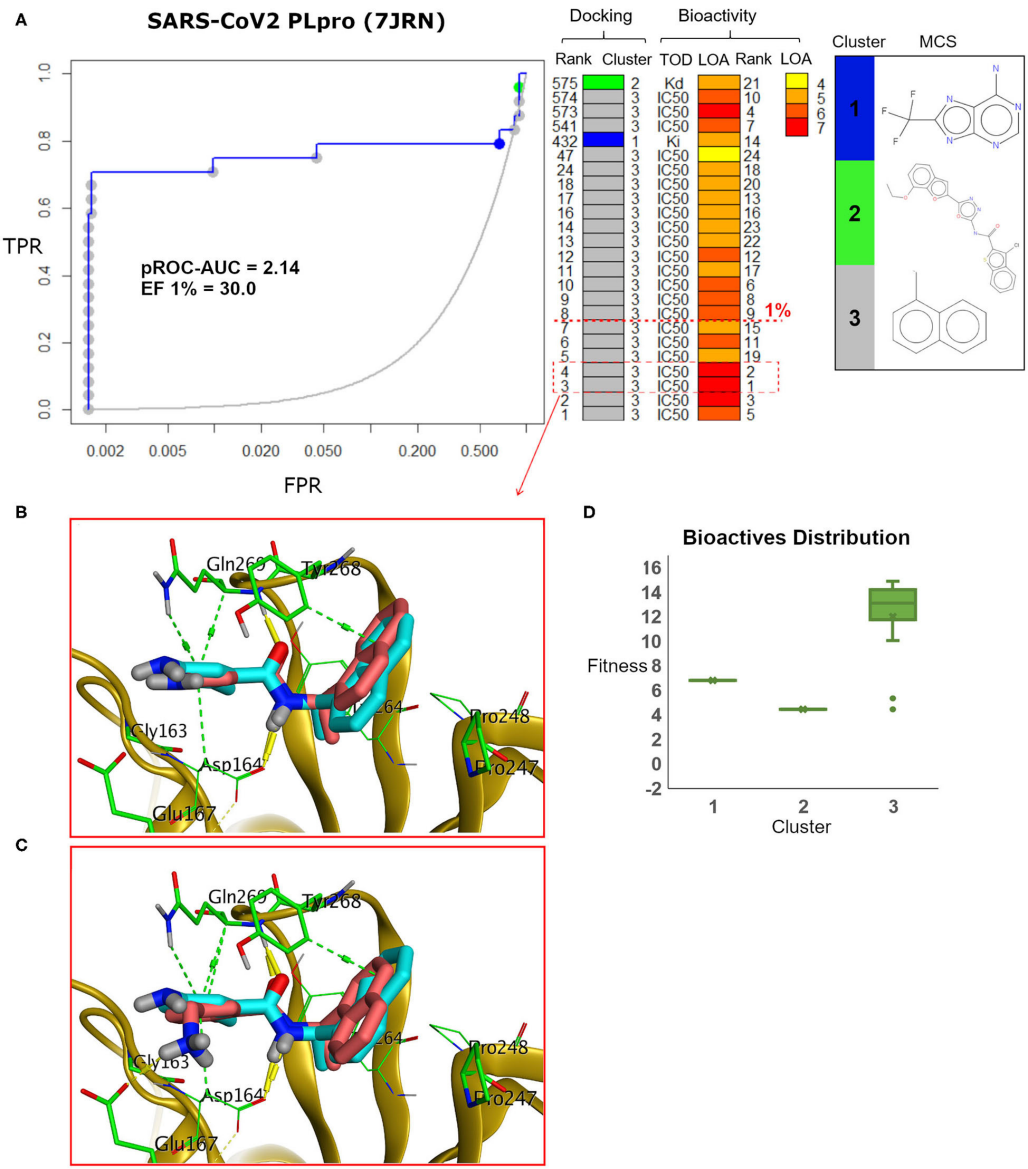
**(A)** pROC-Chemotype plot of the recently introduced SARS-CoV-2 PLpro (PDB ID: 7JRN) using the FRED docking tool. The docking information is matched with the chemotype represented by the cluster number and the bioactivity information. **(B,C)** The docking poses of the two best bioactive molecules (i.e., bioactivity rank 1 and 2) overlaid on the co-crystal ligand as salmon and cyan sticks, respectively. **(D)** Box plot of the fitness vs. chemotype clusters illustrating the bioactive molecule distribution.

The pROC-Chemotype plot of the recently introduced X-ray SARS-CoV2 complexed with **TTT** (PDB ID: 7JRN) for FRED docking displayed comparable results to the homology model, as seen in Figure 9. Both protein structures exhibited similar pROC-AUC and EF 1% values. Additionally, similar bioactive molecules (6 out of 7 molecules) were enriched at EF 1% for both protein structures. Also, the best two bioactive compounds in the bioactive set (i.e., with bioactivity rank 1 and 2) exhibited similar poses in the binding site (Figures 9B,C) compared to their respective poses in the homology model (Figures 7C,B). The docking fitness distribution of the bioactive compounds in this case (Figure 9D) appeared to be related to their distribution in the case of the homology model (Figure 7D). Generally, such behavior is not surprising since both the X-ray SARS-CoV2 PLpro (PDB ID: 7JRN) and the model protein structures exhibit similar conformations for the key residues of the binding site, as discussed earlier (see Figure 5).

### Virtual Screening of the DrugBank Database

These promising benchmarking outcomes encouraged us to employ FRED in a virtual screening campaign to screen the FDA-approved drugs from the DrugBank (Wishart et al., 2018) database against SARS-CoV-2 PLpro. We used the homology model, the X-ray co-crystal structure with a peptide inhibitor (PDB ID: 6WUU), as well as the recently introduced X-ray structure complexed with **TTT** (PDB ID: 7JRN). We utilized these three structures as an approach to target diverse conformations of the ligand-bound state of the binding site and to extract consensus ranking of the screened drugs. The results of the best enriched 1% of the DrugBank database are shown in Table 1.

**Table 1:** The best-ranked 1% of the VS efforts for FDA-approved drugs (DrugBank—release March 2020) against the SARS-CoV-2 PLpro homology model, the co-crystal structure (PDB ID: 6WUU), and the recently introduced co-crystal structure (PDB ID: 7JRN) for (A–C), respectively.

**Docking rank**	**Drug^a^**	**Docking score^b^**	**Molecular weight**	**DrugBank ID**	**Status**
**(A) SARS-CoV2 PLpro (MODEL)**					
1	Perphenazine	−12.94	404.0	DB00850	Approved
2	Zuclopenthixol	−12.57	401.0	DB01624	Approved; investigational
3	Benznidazole	−12.51	260.3	DB11989	Approved; investigational
4	Acetohexamide	−12.08	324.4	DB00414	Approved; investigational; withdrawn
5	Metoclopramide	−11.65	299.8	DB01233	Approved; investigational
6	Tolazamide	−11.54	311.4	DB00839	Approved; investigational
7	Chlorpropamide	−11.53	276.7	DB00672	Approved; investigational
8	Periciazine	−11.52	365.5	DB01608	Approved; investigational
9	Pantothenic acid	−11.25	219.2	DB01783	Approved; nutraceutical; vet_approved
10	Dexpanthenol	−11.19	205.3	DB09357	Approved
11	Agomelatine	−11.11	243.3	DB06594	Approved; investigational
12	Lomustine	−11.11	233.7	DB01206	Approved; investigational
13	Isocarboxazid	−11.04	231.3	DB01247	Approved
14	Practolol	−10.96	266.3	DB01297	Approved
15	Vaborbactam	−10.95	297.1	DB12107	Approved; investigational
16	Salsalate	−10.94	258.2	DB01399	Approved
17	Erdosteine	−10.93	249.3	DB05057	Approved; investigational
18	Sulpiride	−10.79	341.4	DB00391	Approved; investigational
19	Cephalexin	−10.79	347.4	DB00567	Approved; investigational; vet_approved
20	**Midodrine^c^**	−10.79	254.3	DB00211	Approved
21	Nadolol	−10.76	309.4	DB01203	Approved
22	Fluphenazine	−10.74	437.5	DB00623	Approved
23	Acetophenazine	−10.71	411.6	DB01063	Approved
24	Paroxetine	−10.68	329.4	DB00715	Approved; investigational
25	**Benserazide**	−10.56	257.2	DB12783	Approved; investigational
Average (SD)^d^ = −11.26 (±0.64)					
**(B) SARS-CoV2 PLpro (PDB ID: 6WUU)**					
1	**Benserazide**	−10.12	257.2	DB12783	Approved; investigational
2	5-O-Phosphono-alpha-D-ribofuranosyl diphosphate	−10.01	390.1	DB01632	Approved; experimental; investigational
3	Omeprazole	−9.77	345.4	DB00338	Approved; investigational; vet_approved
4	N-Acetylglucosamine	−9.73	221.2	DB00141	Approved; investigational; nutraceutical
5	Losartan	−9.57	422.9	DB00678	Approved
6	Melatonin	−9.50	232.3	DB01065	Approved; nutraceutical; vet_approved
7	**Midodrine**	−9.34	254.3	DB00211	Approved
8	Pyrophosphoric acid	−9.15	178.0	D04160	Approved; experimental
9	Lactulose	−9.12	342.3	DB00581	Approved
10	Mycophenolic acid	−9.06	320.3	DB01024	Approved
11	Glasdegib	−8.99	374.4	DB11978	Approved; investigational
12	Unoprostone	−8.87	382.5	DB06826	Approved; investigational
13	Calcium glucoheptonate	−8.78	490.4	DB00326	Approved
14	Magnesium gluconate	−8.70	450.6	DB13749	Approved; investigational
15	Calcium gluconate	−8.70	430.4	DB11126	Approved; vet_approved
16	Potassium gluconate	−8.70	234.2	DB13620	Approved
17	Ferrous gluconate	−8.70	446.1	DB14488	Approved
18	Chromium gluconate	−8.70	637.4	DB14528	Approved
19	Copper gluconate	−8.70	453.8	DB11246	Approved; investigational
20	Zinc gluconate	−8.70	455.7	DB11248	Approved; vet_approved
21	Aminohippuric acid	−8.66	194.2	DB00345	Approved; investigational
22	Mannitol busulfan	−8.57	338.3	DB12097	Approved; investigational
23	Tipiracil	−8.53	242.7	DB09343	Approved; investigational
24	Indacaterol	−8.53	392.5	DB05039	Approved
25	Naftazone	−8.52	215.2	DB13680	Approved
Average (SD)^d^ = −9.03 (±0.49)					
**(C) SARS-CoV2 PLpro (PDB ID: 7JRN)**					
1	Isocarboxazid	−11.84	231.3	DB01247	Approved
2	Procainamide	−11.68	235.3	DB01035	Approved
3	Metoclopramide	−11.50	299.8	DB01233	Approved; investigational
4	Sulpiride	−11.44	341.4	DB00391	Approved; investigational
5	**Benserazide**	−11.41	257.2	DB12783	Approved; investigational
6	Erdosteine	−11.10	249.3	DB05057	Approved; investigational
7	Pyrophosphoric acid	−11.10	178.0	D04160	Approved; experimental
8	Remoxipride	−11.09	371.3	DB00409	Approved; withdrawn
9	Dexpanthenol	−10.99	205.3	DB09357	Approved
10	**Midodrine**	−10.98	254.3	DB00211	Approved
11	Agomelatine	−10.88	243.3	D06594	Approved; investigational
12	Dobutamine	−10.49	301.4	DB00841	Approved
13	Pantothenic acid	−10.36	219.2	DB01783	Approved; nutraceutical; vet_approved
14	Sulfabenzamide	−10.28	276.3	DB09355	Approved
15	5-O-phosphono-alpha-D-ribofuranosyl diphosphate	−10.22	390.1	DB01632	Approved; experimental; investigational
16	Cefadroxil	−10.21	363.4	DB01140	Approved; vet_approved; withdrawn
17	Nialamide	−10.16	298.3	DB04820	Approved; withdrawn
18	Pergolide	−10.11	314.5	DB01186	Approved; investigational; vet_approved; withdrawn
19	Chlorthalidone	−10.07	338.8	DB00310	Approved
20	Salsalate	−10.03	258.2	DB01399	Approved
21	Pirbuterol	−10.02	240.3	DB01291	Approved
22	Fenoterol	−10.00	303.4	DB01288	Approved; investigational
23	Mefenamic acid	−9.99	241.3	DB00784	Approved
24	Eslicarbazepine acetate	−9.99	296.3	DB09119	Approved
25	Tolnaftate	−9.96	307.4	DB00525	Approved; investigational; vet_approved
Average (SD)^d^ = −10.64 (±0.62)					

a *Drug: is the generic name of the drug*.

b *Docking score is expressed as “FRED Chemgauss4 score”*.

c *Consensus drugs resulting from both protein structures VS are bold-formatted*.

d *Average and standard deviation (SD) are for the docking scores*.

As a consensus, both the model and the X-ray structure complexed with **TTT** (PDB ID: 7JRN) enriched similar 10 out of 25 drugs at 1% of the DrugBank database, as seen in Tables 1A,C. However, only 2 drugs out of 25 drugs were enriched together for the model and the X-ray structure complexed with peptide-like inhibitor (PDB ID: 6WUU), as shown in Tables 1A,B. Interestingly, as a consensus for all the three SARS-CoV2 PLpro structures, two drugs appeared to be commonly enriched, namely: Benserazide and Midodrine. However, the latter is in its prodrug form and therefore is not considered in our investigation.

It is worth mentioning that Perphenazine, Benserazide, and Isocarboxazid appeared to be the best-ranked drugs for the three SARS-CoV2 PLpro structures: the model, PDB ID: 6WUU and PDB ID: 7JRN, respectively.

**Figure 10:**
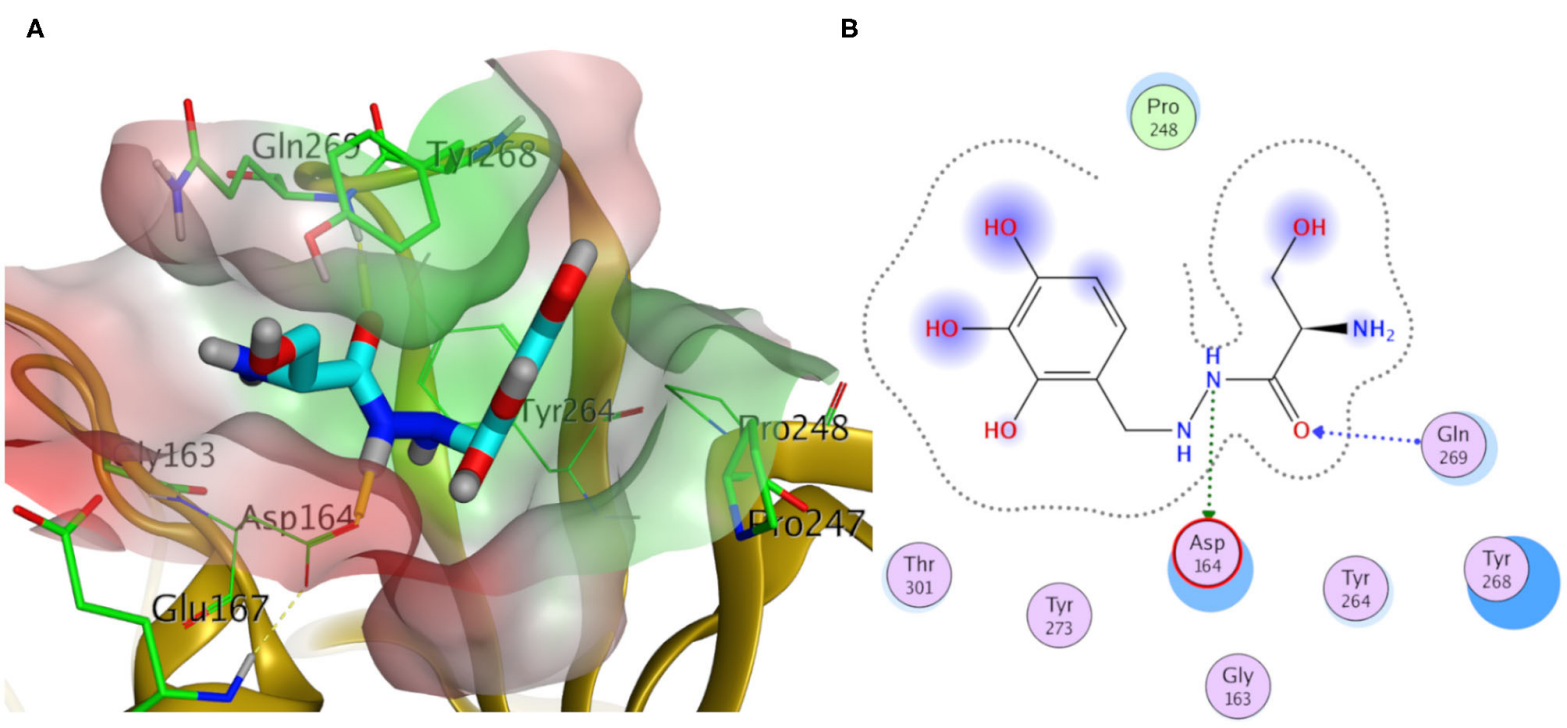
Docking pose of benserazide as cyan sticks in the binding site of SARS-CoV-2 PLpro (PDB ID: 7JRN) in three- and two- dimensional depictions for **(A,B)**, respectively. Polar and non-polar regions of the binding site were presented by red- and green-colored molecular surfaces, respectively. Dashed lines indicate favorable interactions. Non-polar hydrogen atoms were omitted for clarity.

Elucidating the postulated binding interactions of a consensus binder from the DrugBank to the three PLpro protein structures, Figure 10 shows the binding pose of Benserazide in the binding site of the X-ray SARS-CoV2 PLpro structure (PDB ID: 7JRN). Benserazide is a decarboxylase inhibitor usually combined with levodopa to treat Parkinson's disease. Also, benserazide has been conferred by European Medicines Agency as an orphan designation since 2015 for its potential to be used as a therapy for beta thalassaemia. It was marketed since 1977 by Hoffmann La Roche. Its postulated binding pose in the SARS-CoV-2 PLpro binding site exhibited H-bonding interactions via its hydrazide group with side chains of Asp164 and the key residue Gln296, as seen in Figure 10. Also, its trihydroxy phenyl group appeared to be packed in the hydrophobic cleft (green surface in Figure 10A) formed by the key residue Tyr268 with residues Pro247 and Pro248. It is worthy to mention that this binding pose of Benserazide is reproduced for the homology model, while differences were observed for 6WUU (data not shown). Again, this is not surprising due to the high similarity of key residues conformations between the model and 7JRN.

## Conclusion

MSA and protein structure superposition revealed high sequence identity between SARS-CoV PLpro and SARS-CoV-2 PLpro with 82.9 and 100% identity for the binding site. The key residues Tyr269 and Gln270 of the binding site of SARS-CoV PLpro for small-molecule recognition are also present in SARS-CoV-2 PLpro. This encouraged us to use the reported small-molecule binders to SARS-CoV PLpro to generate a high-quality DEKOIS 2.0 benchmark set. Accordingly, we performed a cross-benchmarking study using the SARS-CoV PLpro benchmark set against SARS-CoV-2 PLpro. There is no reported co-crystal structure of SARS-CoV-2 PLpro with the conventional small-molecule inhibitor; hence, there is a lack of information for the binding site in a ligand-protein conformation. Thus, we built a homology model for SARS-CoV-2 PLpro complexed with a small-molecule ligand for benchmarking and docking purposes. Three publicly available docking tools were employed in the benchmarking study against the model, FRED, AutoDock Vina, and PLANTS. All showed better-than-random performances with pROC-AUC values of 2.34 for FRED, compared to pROC-AUC values of 1.35 and 0.98 for AutoDock Vina and PLANTS, respectively. Visualizing the FRED performance via the pROC-Chemotype plot emphasizes that this docking tool can enrich the best bioactivity in the early docking rank. Cross-benchmarking against the X-ray co-crystal structure with a peptide-like inhibitor (PDB ID: 6WUU) confirmed that FRED is the best-performing tool. Furthermore, we performed cross-benchmarking against the recently introduced X-ray structure complexed with a small-molecule ligand (PDB ID: 7JRN). Interestingly, its screening performance and chemotype enrichment were comparable to the built model signifying the high quality of the built model. This encourages us to employ FRED in a VS campaign using the FDA-reported drugs (from DrugBank) against SARS-CoV-2 PLpro. In general, this study offers an example of how to employ a DEKOIS 2.0 benchmark set against a vital target of SARS-CoV-2. This can help improve the success rate for many virtual screening campaigns against the rapidly resolved protein structures of SARS-CoV-2, for fighting the quickly emerging COVID-19.

## Methods

### Multiple-Sequence Alignment and Homology Modeling

The protein sequences of SADS, MERS, SARS-CoV-2, and SARS-CoV PLpro were retrieved as FASTA format from the Protein Data Bank (PDB) using the PDB IDs: 6L5T, 5W8U, 6W9C, and 2FE8, respectively. The multiple sequence alignment is performed using Clustal Omega (Sievers et al., 2011) and presented by ESpript v3.0 (Robert and Gouet, 2014) web server.

SWISS-MODEL (Waterhouse et al., 2018) web server is used to build a homology model for the small-molecule-bound conformation of SARS-CoV-2 PLpro using its automated mode. The template (PDB ID: 3E9S, chain A for SARS-CoV PLpro) was the best recommended for ligand-bound conformation via quality estimate metrics of SWISS-MODEL (Benkert et al., 2011; Waterhouse et al., 2018). The template X-ray crystal structure is with 2.5 Å resolution and R-value free of 0.261. The small-molecule co-crystal ligand is with chemical name “5-amino-2-methyl-N-[(1R)-1-naphthalen-1-ylethyl]benzamide” and involved in the bioactive set for benchmarking with bioactivity rank 1 and IC50 value 230 nM (Lee et al., 2015). This small molecule is included in the built homology model. The Ramachandran plot of SWISS-MODEL was used to test the validity of the model. Furthermore, the structure analysis and verification server (SAVES, 2020) of the University of California Los Angles (UCLA) is used to assess the model, using PROCHECK (Laskowski et al., 1993), Verify 3D (Bowie et al., 1991), PROVE (Pontius et al., 1996), and ERRAT (Colovos and Yeates, 1993).

### Benchmarking and Virtual Screening

#### Preparation of Protein Structures

Molecular Operating Environment (MOE) was used to prepare the protein structures for docking experiments, including (i) the homology model complex of SARS-CoV-2 PLpro, (ii) the apo forms of SARS-CoV-2 PLpro (PDB ID: 6W9C) and SARS-CoV PLpro (PDB ID: 2FE8), (iii) the co-crystal structure of SARS-CoV PLpro (PDB ID: 3E9S), (iv) the co-crystal structure SARS-CoV2 PLpro (PDB ID: 6WUU), and (v) the recently introduced co-crystal structure SARS-CoV2 PLpro (PDB ID: 7JRN). Module “Quickprep” of MOE was used at default settings after removing the redundant chains, irrelevant ions, molecules of crystallization, and solvent atoms (if any). Briefly, these settings include using the “Protonate 3D” function to optimize the H-bonding network and allow ASN/GLN/HIS to flip during protonation. Also, these settings involve refining the ligand and binding site atoms via energy minimization to an RMS gradient of 0.1 kcal/mol/A, while a force constant (strength = 10) was applied for the restraints of receptor atoms. The rest of the receptor atoms outside the binding site were kept fixed. These settings produced a non-significant change of the binding site/ligand coordinates. Also, none of the HIS residues were inspected in the binding site which can be affected by certain protonation/tautomerization state. Conformations of GLN and ASN can be depicted in the respective Figures (in the Results and Discussion section) of the binding site. The prepared structures were saved as mol2 for docking experiments.

#### Preparation of the DEKOIS 2.0 Benchmark Set and DrugBank-Approved Drugs

The DEKOIS 2.0 (Bauer et al., 2013) protocol was applied on 24 SARS-CoV PLpro bioactives, which were extracted from BindingDB, to generate 720 challenging decoys (1:30 ratio). Then, all molecules were prepared by MOE with comparable settings to the previous report (Bekhit et al., 2019). Only one conformer was retrieved, and one protonation state was generated at pH 7.0 for each molecule. The specified stereo configuration of all bioactives, decoys, and DrugBank molecules was retained. All prepared molecules were saved as SD files. The SD files were converted and split into PDBQT files by OpenBabel (O'Boyle et al., 2011) for AutoDock Vina docking experiments and into mol2 files for PLANTS docking experiments.

#### Docking Experiments

For AutoDock Vina (version 1.1.2) (Trott and Olson, 2010) docking, the protein files were converted to PDBQT files by employing a python script (*prepare_receptor4.py*) provided by the MGLTools package (version 1.5.4) (Sanner, 1999). The search efficiency of the docking algorithm was kept at default level, while the size of the docking grid was 22.5 Å × 22.5 Å × 22.5 Å, with a grid spacing of 1 Å to make sure to cover all geometries of the docked compounds. For PLANTS (Korb et al., 2009) docking, the scoring function used was “ChemPLP,” with the “screen” mode selected. The binding site was defined within 5 Å of the coordinates of the complexed ligand, and the apo structures were superposed on the complexed ones to extract similar binding site surroundings. For the OEDocking v3.2.0.2 docking (McGann, 2011, 2012), the FRED docking module (McGann, 2011, 2012) was used at default settings. MakeReceptor GUI of OpenEye was used to define the binding site as a search box around the complexed ligand with 19.69 Å × 16 Å × 15.67 Å dimensions.

#### pROC Calculations

The docking rank was used in calculating the pROC-AUC employing “R-Snippet” component of KNIME (Berthold et al., 2007) according to the following equation (Clark and Webster-Clark, 2008):

$$\begin{array}{l} pROC\;AUC=\frac{1}{n}\;\overset{n}{\underset{i}{\sum }}\left[{-log}_{10}\left(D_{i}\right)\right]=\frac{1}{n}\overset{n}{\underset{i}{\sum }}\log_{10}\left(\frac{1}{D_{i}}\right) \end{array}$$

where *n* is the number of bioactives and *Di* is the fraction of decoys ranked higher than the *ith* bioactive found.

The pROC-Chemotype plots were generated by the “pROC-Chemotype plot” tool which is available in http://www.dekois.com/ (Ibrahim et al., 2014, 2015b).

Protein structure Figures were rendered using Pymol^2^ and MOE.

## Data Availability Statement

The SARS-CoV PLpro active and decoy sets (DEKOIS 2.0 set) can be found in the Supplementary Material. The rest of raw data supporting the conclusions of this article will be made available by the authors, without undue reservation.

## Author Contributions

TI designed the experiments. TI and MI carried out all experiments. All authors have given approval to the final version of the manuscript and agree to be accountable for the content of the work.

## Conflict of Interest

MB was employed by the company AstraZeneca (Cambridge, UK). The remaining authors declare that the research was conducted in the absence of any commercial or financial relationships that could be construed as a potential conflict of interest.

## Footnotes

^2^The PyMOL Molecular Graphics System, Schrödinger, LLC.

## Supplementary Material

Supplementary Table 1 The active set of SARS-CoV PLpro.

Supplementary Table 2 The challenging DEKOIS 2.0 decoy set.

## Abbreviations

WHO: World Health Organization
COVID-19: corona virus disease 19
SARS-CoV-2: severe acute respiratory syndrome coronavirus 2
ssRNA: single stranded RNA genome
MERS-CoV: middle east respiratory syndrome coronavirus
PLpro: papain-like protease
3CLpro: 3C-like protease
Mpro: main protease
DEKOIS: Demanding Evaluation Kits for Objective *In silico* Screening
VS: virtual screening.
